# Availability, prices and affordability of UN Commission’s lifesaving medicines for reproductive and maternal health in Uganda

**DOI:** 10.1186/s40545-017-0123-9

**Published:** 2017-11-15

**Authors:** Denis Kibira, Freddy Eric Kitutu, Gemma Buckland Merrett, Aukje K. Mantel-Teeuwisse

**Affiliations:** 1 WHO Collaborating Centre for Pharmaceutical Policy and Regulation, Utrecht Institute for Pharmaceutical Sciences (UIPS), Utrecht University, Universiteitsweg 99, 3584 CG Utrecht, the Netherlands; 2 Coalition for Health Promotion and Social Development (HEPS-Uganda), Plot 351A, Balintuma Road, Namirembe Hill, Kampala, Uganda; 3 Makerere University, School of Public Health and Pharmacy Department, College of Health Sciences, PO Box 7072, Kampala, Uganda; 4 Uppsala University, Department of Women’s and Children’s Health, International Maternal and Child Health, SE-751 85 Uppsala, Sweden; 5 Health Action International, Overtoom 60 (2), 1054 HK Amsterdam, The Netherlands; 6 WHO Collaborating Centre for Pharmaceutical Policy and Regulation, Utrecht Institute for Pharmaceutical Sciences (UIPS), Utrecht University, Universiteitsweg 99, 3584 CG Utrecht, the Netherlands

## Abstract

**Background:**

Uganda was one of seven countries in which the United Nations Commission on Life Saving Commodities (UNCoLSC) initiative was implemented starting from 2013. A nationwide survey was conducted in 2015 to determine availability, prices and affordability of essential UNCoLSC maternal and reproductive health (MRH) commodities.

**Methods:**

The survey at health facilities in Uganda was conducted using an adapted version of the standardized methodology co-developed by World Health Organisation (WHO) and Health Action International (HAI). In this study, six maternal and reproductive health commodities, that were part of the UNCoLSC initiative, were studied in the public, private and mission health sectors. Median price ratios were calculated with Management Sciences for Health International Drug Price Indicator prices as reference. Maternal and reproductive health commodity stocks were reviewed from stock cards for their availability for a period of 6 months preceding the survey. Affordability was measured using wages of the lowest paid government worker.

**Results:**

Overall none of the six maternal and reproductive commodities was found in the surveyed health facilities. Public sector had the highest availability (52%), followed by mission sector (36%) and then private sector had the least (30%). Stock outs ranged from 7 to 21 days in public sector; 2 to 23 days in private sector and 3 to 27 days in mission sector. During the survey, maternal health commodities were more available and had less number of stock out days than reproductive health commodities. Median price ratios (MPR) indicated that medicines and commodities were more expensive in Uganda compared to international reference prices. Furthermore, MRH medicines and commodities were more expensive and less affordable in private sector compared to mission sector.

**Conclusion:**

Access to MRH commodities is inadequate in Uganda. Maternal health commodities were more available, cheaper and thus more affordable than reproductive health commodities in the current study. Efforts should be undertaken by the Ministry of Health and stakeholders to improve availability, prices and affordability of MRH commodities in Uganda to ensure that sustainable Development Goals are met.

## Background

Maternal mortality is a major public health concern in Uganda. In 2016, Uganda’s maternal mortality was estimated at 336deaths per 100,000 live births [1]. Judged against the Millennium Development Goal 5, Uganda did not achieve the 75% reduction in maternal mortality from the 1990 levels by 2015. [2]. Most of these maternal deaths are associated with events directly related to pregnancy and child birth, such as unsafe abortion and obstetric complications, severe bleeding, infections, pre-eclampsia and obstructed labour, and the proportion of deaths among women of reproductive age that are due to maternal causes is 13.4% [3]. Additionally, pregnancy increases the risk of maternal death from causes of malaria, diabetes, hepatitis, anaemia and HIV/AIDS. Indeed, 3.1% percentage of HIV/AIDS deaths is related indirectly to maternal causes [4]. Studies have shown that these deaths could have been averted if there was adequate access to maternal and reproductive health services [5–7].

The state of sexual reproductive health remains poor in Uganda with a high fertility rate of 5.8 children per woman of child bearing age [1], high rates of teenage pregnancies (24%) [8] and unsafe abortions accounting for 11% of maternal deaths annually [8]. In addition, there is limited demand for, and uptake of, reproductive health services, with only 20.4% of Ugandan women using a modern contraceptive method. The Contraceptive Prevalence Rate (CPR) stands at 30% and the unmet family planning need stands at 28% [9]. This situation is exacerbated by supply chain bottlenecks that impair the last mile delivery [10].

In 2010 the UN General Secretary launched the Every Woman Every Child (EWEC) movement to address challenges and bottlenecks to reduction of maternal and child mortality. The preceding review to the EWEC movement had identified unavailability and inadequate access to proven life-saving low-cost medicines and commodities. Therefore, the UN Commission on Life Saving Commodities (UNCoLSC) identified and highlighted 13 underused, low-cost and high impact medicines and medical devices for reproductive, maternal, new-born and child health with the greatest potential to reduce preventable deaths [11]. It also proposed mechanisms to increase the availability, adequate access and rational use of the 13 identified life-saving commodities.

Given the poor progress towards achieving MDG goal 5, Uganda received technical and financial support to conduct a reproductive, maternal, new born and child health (RMNCH) situation analysis to inform the development of an evidence-based country specific implementation plan [8]. Following a two-year implementation period, a nationwide survey was conducted to determine the availability and prices of the six maternal and reproductive health commodities from among the UNCoLSC commodities within the public, private and mission sectors. Additionally, this study also determined the stock out duration for the same basket of commodities to provide information on how fast the system responds to stock outs.

## Methods

A survey measuring the availability, price and affordability of maternal and reproductive health (MRH) commodities at health facilities in Uganda was conducted in September 2015, using an adapted World Health Organisation (WHO) and Health Action International (HAI) standardized methodology [12]. This method was validated [13] and used by others [14–16]. It is based on quantitative techniques to analyse availability and prices of health commodities in the public, private and mission health sectors.

Public, private and mission sector health centres of level III or higher participated in the survey.

MRH commodity availability on day of survey and in 6 months preceding the survey was assessed and prices paid by patient were collected.

### Selection of outlets

The central region which is the largest in the country and has the capital city was selected first. Three regions of Eastern, Western and Northern Uganda within 1 day’s travel from the central region were then selected to provide a realistic representation of the diverse epidemiological, geographical and medicine supply chain characteristics in Uganda. Health facilities from both urban and rural areas were included in the study sample.

In each region, the main regional referral hospitals were selected with guidance of the Uganda Ministry of Health list of health facilities; public health centres level III or higher were randomly selected. Then private and mission sector health facilities that were within a three-hour drive radius from the enrolled regional referral hospitals were selected, respectively. Consecutive sampling was done with an intention of having 10 health facilities per sector in each region coming up to a total sample frame of 120 facilities. This was done to ensure that each sector had a minimum representation of 30 health facilities in the survey [12]. Health Centres level III are the lowest level of care at which MRH commodities are delivered according to the Ministry of Health (MoH) scheduling of basic of health services in Uganda [17].

### Selection of medicines and commodities

The medicines and commodities surveyed included the six reproductive and maternal health medicines and commodities, which are required either to prevent or manage pregnancy as specified by the “United Nations Commission on Life Saving Commodities for Women and Children” (UNCoLSC). UNCoLSC prioritized a core list of 13 life-saving commodities and medicines for reproductive, maternal, newborn and child health, and it specified their formulation or presentation. All countries, Uganda inclusive, were encouraged to grant marketing authorization to these medicines and commodities. The final list of products measured is shown in Table 1 below.

**Table 1:** List of medicines and commodities surveyed in Uganda, 2015, based on the standard World Health Organization/Health Action International Medicine Prices and Availability methodology

Medicine	
Reproductive Health	Use
1- Female condom (any brand)	Contraception
2- Contraceptive implants: a. Etonogestrel 68 mg/rod (Implanon) OR b. Levonorgestrel 0.75 mg/rod (Jadelle)	Contraception
3- Emergency contraceptive pill: a. Levonorgestrel (1.5 mg or 0.75 mg) tablet	Emergency contraception
Maternal Health	
4- Oxytocin injection 10 IU, 1 ml	Prevention and management of post-partum Haemorrhage
5- Misoprostol 200 μg tablet	Prevention and management of post-partum Haemorrhage
6- Magnesium sulphate 500 mg/ml injectable (2 ml, 5 ml, 10 ml ampoule)	Management of pre-eclampsia and eclampsia

### Data collection and analysis

Eight data collectors with previous experience of conducting medicine surveys worked in pairs of a pharmacist and a social scientist under close supervision of a qualified survey manager. Prior to data collection, these pairs were trained on the WHO/HAI methodology of monitoring medicine availability and prices. Data collectors used a semi-structured questionnaire to interview facility managers while ascertaining physical count and stock card records of surveyed medicines. Availability was measured by the physical presence of a product in the outlet at the time of the survey. For each medicine surveyed, data collectors recorded the stated product name for both the highest and lowest priced medicines available, the manufacturer, unit price of the product and number of stock-out days in the previous 6 months. In the public sector where medicines are free of charge to the care seekers, only availability and stock out days were recorded.

Once data collection was complete, survey data was entered centrally into the pre-programmed Microsoft Excel Workbook provided as part of the WHO/HAI methodology. Data input was independently checked for errors. Additional quality control measures were executed at various stages throughout the study. An advisory team provided the overall quality assurance by reviewing survey process, tools for data collection and validation of findings. The survey tools were pre-tested before the survey and prior to data collection. In addition, all survey personnel participated in training and field testing of the survey. Each regional/district team had a supervisor who cross checked the data on a daily basis for completeness, legibility and consistency and reported to the survey manager. A survey manager made field visits and follow-up telephone interviews to validate data in 10% of the sampled outlets. Prior to data entry all relayed data was checked for completeness and consistency.

The availability of individual medicines was calculated as the percentage of sampled medicine outlets where the medicine was found. Data were reported in aggregate as public, private or mission sector medicine outlets. Overall availability per sector was calculated as median of medicines surveyed. For stock data, facilities that had not stocked a particular medicine for 6 months preceding the survey were expressed as a percentage of total number of facilities. For those that reported to stock the medicine, a monthly average of stock-out days was calculated.

Patient prices were collected in Uganda Shillings and the median, minimum and maximum unit prices were estimated. To facilitate cross-country comparisons, medicine prices obtained during the survey were expressed as ratios relative to a standard set of international reference prices [18] by dividing the median local unit price by the international reference unit price. Medicine price ratios were calculated only for medicines with price data from at least four medicine outlets. The exchange rate used to calculate MPRs was 1$ = 3667.9 Uganda Shillings; this was the mid-rate (average of purchase and sale rate) taken from Bank Uganda website on the first day of data collection [19].

Affordability was calculated using the number of days it requires to pay for standard treatment or dose of treatment based on the daily income of the lowest-paid unskilled government employee [12]. The daily wage of the lowest paid government worker (attendants) is approximately UGX 6255 (USD 1.78) as per Uganda Ministry of Public Service salary structure [20]. Treatments that required more than 1 day’s wages to purchase were considered unaffordable [12].

## Results

A sample of 114 facilities comprising of 37 public, 41 private and 36 mission sector health facilities participated in the study as is shown in Table 2 below.

**Table 2:** Number and distribution of health facilities surveyed

Sector	Central	Eastern	Western	North	Total
Public	10	08	11	08	37
Private	13	09	09	10	41
Mission	11	07	09	09	36
Totals	34	24	29	27	114

### Availability on the day of data collection

**Fig. 1:**
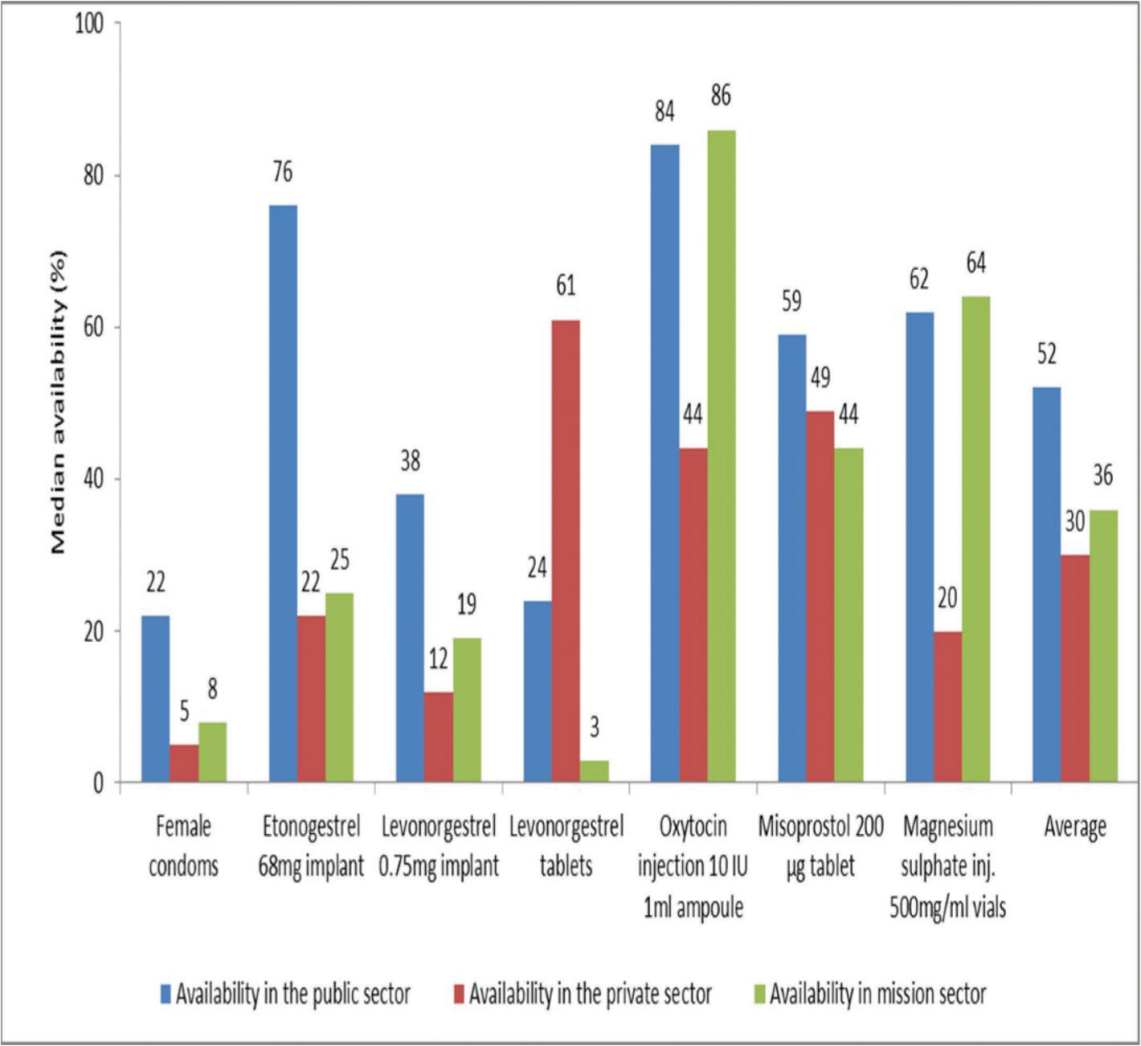
Median availability of reproductive and maternal health medicines and commodities on day of data collection: Figure shows that overall no commodity was found in all facilities. The public sector had the highest availability, followed by mission sector and the private sector had the least availability

Availability of medicines on day of data collection is shown in Fig. 1. Overall none of the maternal and reproductive health commodities studied was found in all the surveyed health facilities. The public sector had the highest median (52%), followed by mission sector (36%) and then private sector had the least (30%). The most available commodity was oxytocin injection (86% in mission facilities and 84% in public facilities). The least available commodity was the female condom (in 5% of private facilities, 8% of mission facilities and 22% of facilities). In the public sector, three out of seven items were available in less than 50% of facilities. In the private sector, six of seven items were available in less than 50% of facilities whereas in mission sector five medicines were available in less than 50% of facilities.

Maternal health commodities were more available than reproductive health commodities. Among reproductive health commodities, the long term contraceptive etonogestrel implant (brand name Implanon) was most available at 76% in public facilities.

### Medicine stock-out duration

**Fig. 2:**
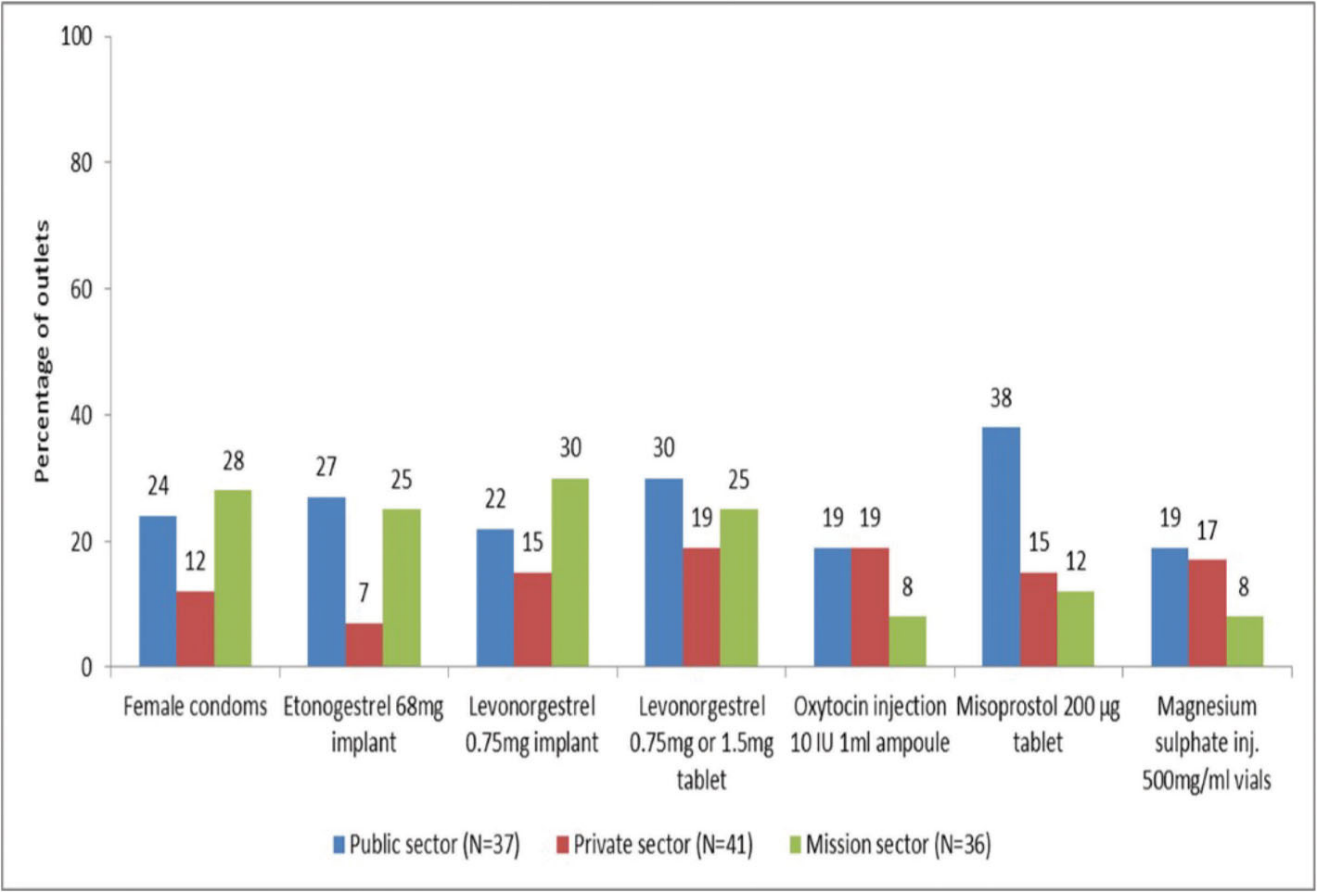
Percentage of surveyed outlets that had not stocked the named reproductive or maternal medicine or commodity in 6 months preceding survey: a large number of facilities had not stocked many of the commodities

During the review period, a large number of facilities (44% public facilities, 49% private facilities, 59% mission facilities on average) had not stocked MRH commodities in the 6 months preceding the survey; 38% public facilities had not stocked misoprostol, 19% private facilities had not stocked levonorgestrel tablets and 30% mission facilities had not stocked levonorgestrel implants (see Fig. 2).

**Fig. 3:**
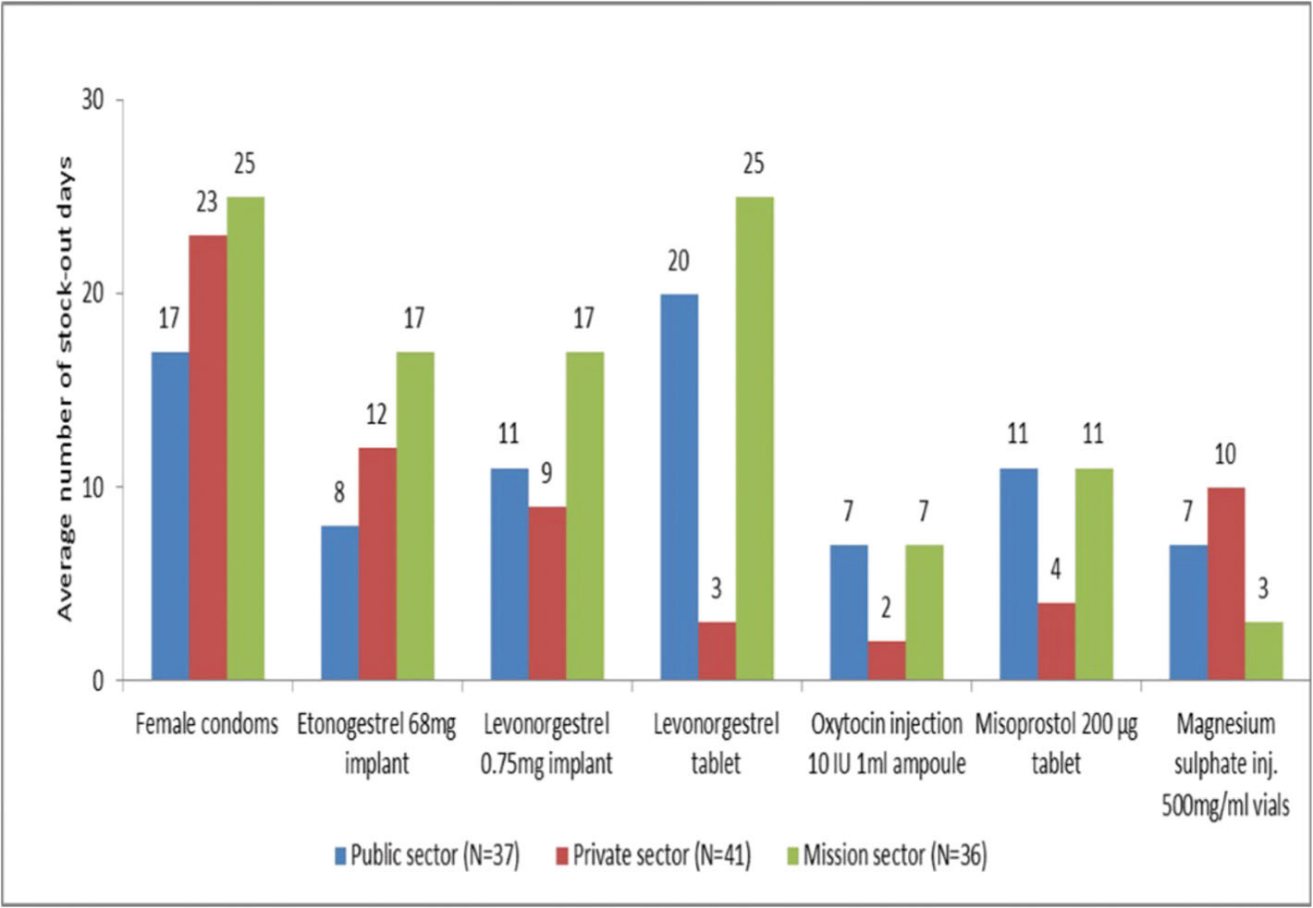
Average number of stock-out days per month of each reproductive and maternal medicine or commodity at surveyed outlets in 6 months preceding survey: Stock out days per month ranged from 7 days to 20 days in the public sector; 2 days to 23 days in the private sector and 3 days to 27 days in the mission sector

For the facilities that had stocked the items in the previous 6 months preceding the survey, the stock out days ranged from 7 days to 20 days in the public sector; 2 days to 23 days in the private sector and 3 days to 27 days in the mission sector, respectively (see Fig. 3). Although ranges of stock out days were similar, pronounced differences existed between sectors for some commodities, e.g. for levonorgestrel tablets. Maternal health commodities had less stock out days in the 6 months preceding the survey than reproductive health commodities. Female condoms were the least stocked commodity across all sectors.

### Prices and affordability of commodities in private and mission sectors

Median price ratios (MPR) indicated that medicines and commodities were up to over four times more expensive in Uganda compared to international reference prices (Table 3). Also medicines and commodities were more expensive and less affordable in the private sector than the mission sector.

**Table 3:** Prices and affordability of treatment

Medicine	Unit Price in USD (MPR)		Treatment unit	Affordability per treatment unit (in days)	
	Priv.	Mission		Private sector	Mission sector
Etonogestrel 68 mg/rod × 1 implant	3.03	–	1 implant	1.6	–
Levonorgestrel 0.75 mg tablet	1.52	–	2 tablets	1.6	–
Misoprostol 200 μg Tablet	1.52 (4.39)	0.91 (2.63)	I tablet	0.8	0.48
Oxytocin Injection 10 IU, 1 ml	0.61 (3.49)	0.61 (3.49)	1 ampoule	0.32	0.32
Magnesium sulfate Injection 500 mg/ml	2.04 (1.54)	1.52 (1.15)	1 ampoule	1.1	0.8

## Discussion

Overall no MRH commodity was available in all the surveyed facilities. Commodities were available in just half of public facilities and in about one third of both mission and private sector facilities. Up to one in three facilities had not stocked many of the MRH commodities in a period of 6 months. Medicines and commodities were more expensive in Uganda than to the international reference prices and were less affordable in private sector than to mission sector.

A core obligation of state as regards the right to reproductive health is to ensure the availability, accessibility, acceptability and quality of services [21]. Essential commodity supplies are required to ensure that healthy reproductive care is made possible. Child bearing individuals have a right to choose, obtain and use contraceptives to avoid unintended pregnancies, to prevent and treat sexually transmitted infections (STIs), and to ensure healthy pregnancy and delivery. This concept is known as *Reproductive Health Commodity Security* (RHCS) and requires governments to ensure and maintain access to and availability of reproductive health commodities [22].

This survey found that universal access to medicines and commodities for reproductive and maternal health has not been achieved in Uganda. Availability was low, stock outs frequently occurred or medicines and commodities had not been stocked during the 6 months preceding the study and they were largely unaffordable because of high prices. This is similar to studies elsewhere; Silal et al. found access to obstetric services in South Africa was impeded by among others availability and affordability barriers and Adjei et al. found low availability of contraceptives in Ghana [5, 23].

Availability of maternal health commodities was better than availability for reproductive health commodities. Between 2012 and the current survey in 2015, availability of reproductive health commodities did not improve but there was an improvement in availability of maternal health commodities in the public sector. For example there was even a reduction in availability of emergency contraceptives from 61% to 24% in the public sector [24]. There was increase in availability of oxytocin in the public sector from 61% to 84% whereas it decreased slightly in the mission sector from 90% to 86% and in the private sector from 86% to 44%. Similarly availability of magnesium sulphate in the public sector improved from 47% to 62% but reduced in mission facilities from 100% to 64%. Availability in the private sector remained minimal consistent with a previous survey [10]. This indicates that management of supplies for family planning programs remains a challenge. The improvement in the public sector may be related to the various government and civil society efforts to improve maternal health in this sector. These campaigns should also be targeted to the other sectors.

The results may indicate limited prioritisation of demand generation activities for reproductive and maternal health commodities by improving knowledge of providers and consumers of the commodities. Policy makers ought to emphasise among others provider skills and overcoming gender inequity and negative social norms to improve access to reproductive and maternal health commodities [25, 26].

Stock outs were high across all sectors but least prone in public sector; on average 63% of public facilities had a stock out in previous 6 months of survey, compared to 80% of mission facilities and 84% of private facilities. However, stock out duration per month was least in the private sector. This implies that the private sector had the most readiness to respond to a stock-out.

Consumer prices for medicines and commodities were very high and unaffordable. For example the “emergency pill” levonorgestrel 0.75 mg had a median unit price of USD 1.52 per tablet and therefore the lowest government worker would have to spend 1.6 days’ wages to afford two tablets required for a dose of treatment. This finding is consistent with many studies done in low and middle income countries which show that medicine prices are often high [27–29]. Efforts should be undertaken by the Ministry of Health and stakeholders like manufacturers, development partners and civil society to reduce commodity prices through measures such as price caps, subsidies, pooled purchasing mechanisms by all sectors and cost-effective strategies to increase the distribution coverage area of wholesalers [30, 31].

The WHO/HAI medicines Prices and Availability survey data can play an important role in analysing access, availability and affordability of essential medicines in low and middle-income countries. The major strength of this study is the use of a tested, reliable, standardized and validated methodology which allows for the measurement of medicine prices and availability [13]. The study provides details on availability, cost, and affordability of individual medicines across three sectors (public, private and mission) and the methodology was adopted to incorporate stock-out rates for the various medicines and commodities and therefore provides a more reliable and accurate picture of availability over a longer period beyond the day of data collection. The study also explored alternative therapeutic alternatives, dosage forms and strengths of the medicines and commodities. Findings in this study may not be generalizable to other countries with pharmaceutical markets and structures markedly different from Uganda’s. However, such information can form an important component of advocacy efforts for rational pharmaceutical policies. In order to provide more useful information for effective policy intervention, and to counter the main limitations of this study, methods to elucidate factors influencing the differences in results between sectors, for example, should be incorporated.

## Conclusions

Results indicate that access to medicines and commodities for reproductive and maternal health has not been achieved in Uganda. Access in terms of availability, prices and affordability was better for maternal health compared to reproductive medicines and commodities. The Ministry of Health therefore ought to emphasise among others, provider skills and overcoming gender inequity and negative social norms to improve access to reproductive and maternal health commodities. Efforts should be undertaken by the Ministry and stakeholders to reduce commodity prices for retailers and other measures such as subsidies, pooled purchasing mechanisms and cost-effective strategies to increase the distribution coverage area of wholesalers.

## Abbreviations

HAI: Health Action International
MoH: Ministry of Health
MPR: Median price ratios
MRH: Maternal and reproductive health
RMNCH: Reproductive, maternal, new born and child health
UNCoLSC: United Nations Commission on Life saving Commodities
WHO: World Health Organisation
